# Inhibition of malic enzyme 1 disrupts cellular metabolism and leads to vulnerability in cancer cells in glucose-restricted conditions

**DOI:** 10.1038/oncsis.2017.34

**Published:** 2017-05-08

**Authors:** S Murai, A Ando, S Ebara, M Hirayama, Y Satomi, T Hara

**Affiliations:** 1 Oncology Drug Discovery Unit, Pharmaceutical Research Division, Takeda Pharmaceutical Company, Kanagawa, Japan; 2 Integrated Technology Research Laboratories, Pharmaceutical Research Division, Takeda Pharmaceutical Company, Kanagawa, Japan

## Abstract

Malic enzyme 1 (ME1) regulates one of the main pathways that provide nicotinamide adenine dinucleotide phosphate (NADPH), which is essential for cancer cell growth through maintenance of redox balance and biosynthesis processes in the cytoplasm. In this study, we found that ME1 inhibition disrupted metabolism in cancer cells and inhibited cancer cell growth by inducing senescence or apoptosis. In glucose-restricted culture conditions, cancer cells increased ME1 expression, and tracer experiments with labelled glutamine revealed that the flux of ME1-derived pyruvate to citrate was enhanced. In addition, cancer cells showed higher sensitivity to ME1 depletion in glucose-restricted conditions compared to normal culture conditions. These results suggest that in a low-glucose environment, where glycolysis and the pentose phosphate pathway (PPP) is attenuated, cancer cells become dependent on ME1 for the supply of NADPH and pyruvate. Our data demonstrate that ME1 is a promising target for cancer treatment, and a strategy using ME1 inhibitors combined with inhibition of glycolysis, PPP or redox balance regulators may provide an effective therapeutic option.

## Introduction

In malignant tumours, oncogenic gene alterations such as point mutations, translocations and gene amplification and deletion are recurrently observed, and the oncogenes and their downstream signals have been targeted for cancer therapy.^1^ In recent decades, it has become clear that cancer cells alter their cellular metabolism to adapt to gene and microenvironment alterations to sustain growth and survival.^2^ Therefore, targeting the reprogramming of cellular metabolism to treat cancers has currently come under the spotlight.^3^

Cancer-associated metabolic reprogramming affects gene expression, cellular differentiation and tumour microenvironment, and these characteristics were recently summarised by Pavlova NN *et al.*^4^ as the six hallmarks of cancer metabolism. One hallmark corresponds to the use of glycolysis and tricarboxylic acid (TCA) intermediates for biosynthesis and nicotinamide adenine dinucleotide phosphate (NADPH) production.

NADPH functions as a reducing agent in cells and has critical roles in macromolecular biosynthesis and redox homoeostasis. NADPH is produced by metabolic enzymes such as glucose-6-phosphate dehydrogenase (G6PD) and 6-phosphogluconate dehydrogenase (6PGD) of the pentose phosphate pathway (PPP), malic enzymes (MEs), isocitrate dehydrogenases (IDHs) and enzymes in one-carbon-tetrahydrofolate oxidation pathways. These NADPH-producing enzymes have been evaluated as potential therapeutic targets of cancer.^5, 6, 7^

MEs regulate cellular energy, redox balance and biomolecular synthesis by converting the TCA cycle intermediate malate into the TCA carbon source pyruvate and NADPH. In mammalian cells, three isoforms of MEs have been identified: a cytosolic NADP^+^-dependent isoform, ME 1 (ME1); a mitochondrial NAD^+^-dependent isoform, ME 2 (ME2); and a mitochondrial NADP^+^-dependent isoform, ME 3 (ME3).^8, 9^ Different from the mitochondrial ME2 and ME3, ME1 produces NADPH in the cytoplasm. It has been reported that ME1 produces NADPH at levels as high as those produced by G6PD in the PPP shunt, which is a major cellular source of NADPH.^10^ ME1 levels are used as a prognosis and predictive marker for radiation therapy in cancer.^11, 12^ Therefore, ME1 is considered a promising target for cancer therapy and has been evaluated as such.^13, 14^

In this study, we explored the use of ME1 as a target of cancer treatment through testing the vulnerability of cancer cells to ME1 inhibition by analysing the resulting alterations in gene expression and metabolite levels.

## Results

### ME1 localises in the cytosol and is expressed in various cancer cell lines

**Figure 1:**
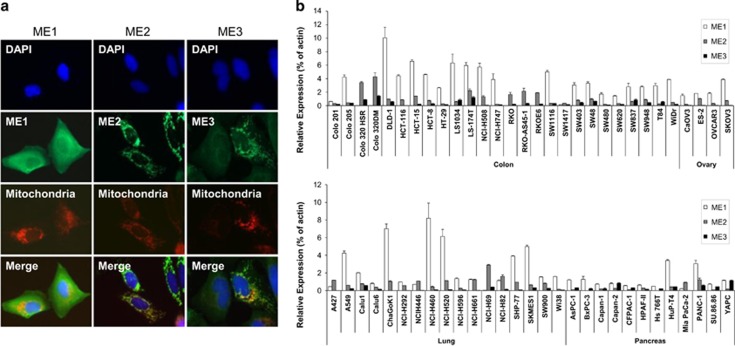
ME1 localises in the cytosol and is expressed in cancer cell lines. (**a**) U2OS cells were transfected with ME1, ME2 or ME3 cDNA plasmid and, 48 h later, cells were fixed and stained with DAPI, anti-ME1, anti-ME2 and anti-ME3 antibodies, and Mito-tracker, which specifically stains mitochondria. Nucleus (blue), ME1, ME2 or ME3 (green), mitochondria (red) and the merged image are shown. (**b**) Basal ME1, ME2 and ME3 mRNA expression levels in colorectal, ovarian, lung and pancreatic cancer cell lines were determined by TaqMan PCR. Expression levels were normalised by expression of β-actin in each cell line and the relative expression is shown in the bar graph (*n*=3).

To identify the localisation of MEs in cancer cells, we conducted immunocytochemistry. U2OS cells were transfected with ME1, ME2 or ME3 cDNAs and immuno-stained with each antibody; mitochondria were stained with Mito-Tracker. As previously reported,^8, 9^ ME1 localised in the cytoplasm, whereas ME2 and ME3 localised in the mitochondria (Figure 1a). We conducted TaqMan analysis to investigate the expression profile of MEs in cells of various cancer types. We found that most cancer cell lines express higher levels of ME1 than of ME2 and ME3, although some cell lines predominantly expressed ME2 and ME3 (Figure 1b). Therefore, we examined the effects of ME1 inhibition on cancer cell proliferation and metabolism.

### ME1 knockdown suppresses cancer cell growth and induces senescence

**Figure 2:**
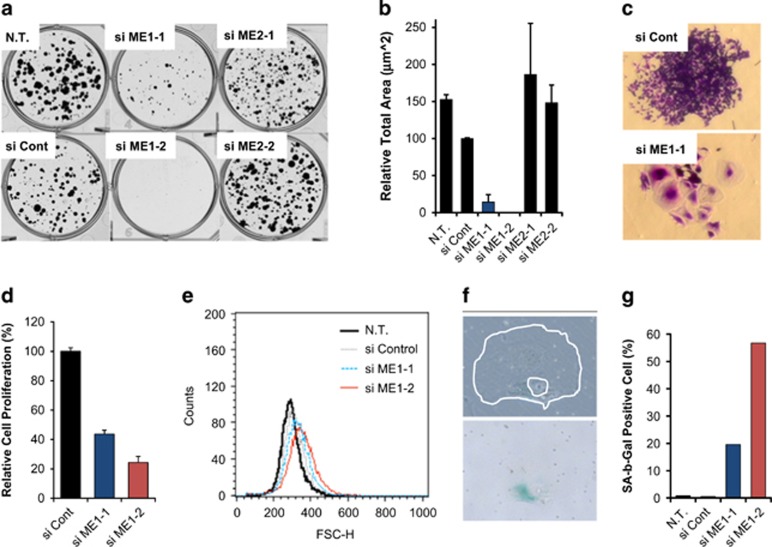
ME1 knockdown induces cellular senescence and suppresses cell growth. (**a**,**b**) HCT116 cells were reversely transfected with control, ME1, or ME2 siRNAs and 1000 cells were seeded on six-well plates. Then, cells were cultured for 11 days for colony formation assay. Colonies were fixed and stained with Crystal violet, and colony area of each well was quantified by GelCount and relative total area is shown as a bar graph (*n*=3, mean with s.d.). (**c**) Light microscopy images of colonies of HCT116 cells transfected with si control and si ME1-1. (**d**) PC3 cells were transfected with ME1 siRNAs and viability was determined 96 h after transfection. Relative cell proliferation is shown as a bar graph (*n*=3, mean with s.d.). (**e**) PC3 cells were transfected with ME1 siRNAs and cultured for 96 h. Cell size forward scatter (FSC) was detected by FACS analysis. (**f**) PC3 cells were transfected with ME1 siRNAs for 9 days and β-galactosidase was then stained. Cell (outer white line) and its nucleus (inner white line) are shown in the upper photograph. β-galactosidase was stained in blue and is shown in the lower photograph. (**g**) β-galactosidase-positive cells were manually counted and the percentage of stain-positive (SA-b-Gal positive) cells are shown in the bar graph. FACS, fluorescence-activated cell sorting.

To examine the effects of ME1 or ME2 inhibition on HCT116 cell growth, we performed knockdown experiments using siRNAs. We excluded ME3 siRNA because ME3 mRNA was not detected in HCT116 cells (Figure 1b). Both ME1 and ME2 mRNA and protein levels were suppressed by siRNAs (Supplementary Figures S1A and S1B). ME1 depletion suppressed colony formation in clonogenic assays (Figures 2b and c), whereas ME2 depletion did not affect HCT116 cell growth. Some colonies of cells transfected with ME1-1 siRNA included enlarged cells (Figure 2d), which is a hallmark of senescent cells.^15^ To confirm these observations, we repeated the experiments with the PC3 cell line. We first examined if ME1 depletion affected PC3 cell growth. ME1 expression was suppressed by the two siRNAs in PC3 cell line (Supplementary Figure S1D). In this condition, we found that ME1 depletion suppressed PC3 cell proliferation 96 h after siRNA reverse-transfection (Figure 2d). Next, we conducted clonogenic assays with the PC3 cell line. PC3 cells were reversely transfected with siRNAs and cultured for nine days. Fluorescence-activated cell sorting analysis revealed that the number of enlarged PC3 cells increased after transfection with ME1 siRNAs (Figure 2e), which is consistent with microscopic observation of colonies (Supplementary Figure S1E). We investigated the expression of SA-b-Gal, which is another hallmark of senescence, in the PC3 colonies and found some SA-b-Gal positive cells (Figure 1f, blue staining). We manually counted SA-b-Gal stain-positive and -negative PC3 cells and calculated the percentage of stained cells. The results show that the number of SA-b-Gal stain-positive cells clearly increased after ME1 knockdown (Figure 1g). These results suggest that ME1 depletion induces cellular senescence and suppresses cancer cell growth.

### ME1 knockdown disturbs malate levels and its downstream metabolism in the HCT116 cell line

**Figure 3:**
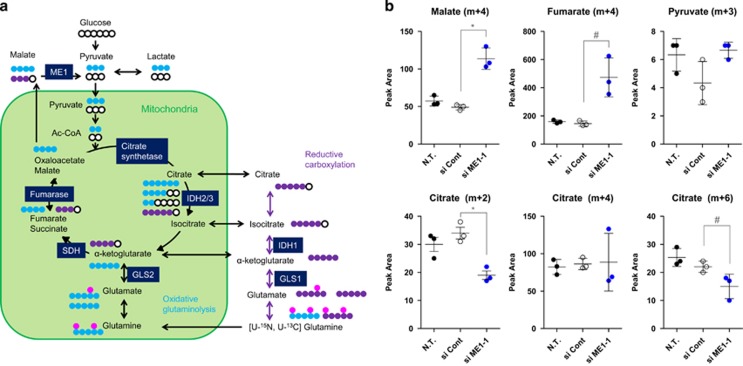
[U-13C, U-15N] l-glutamine isotope profiling analysis revealed malate metabolism is suppressed by si ME-1. (**a**) Schematic representation of glutamine isotope profiling. Blue and purple circles indicate a carbon isotope, metabolized through oxidative glutaminolysis and reductive carboxylation, respectively, pink circles a nitrogen isotope and white circles non-labelled carbons. (**b**) [U-13C, U-15N] l-glutamine profiling analysis was performed in the HCT116 cell line. Cells were cultured for 72 h after siRNA transfection and labelled for 24 h in McCoy's 5A media with 2 mm [U-13C, U-15N] l-glutamine. Cell pellets were then collected for flux analysis. Peak area of each metabolite is shown in the dot graph (*n*=3, *t*-test; *P<0.01; ^#^*P*<0.05).

To examine the metabolic alterations resulting from ME1 depletion, we conducted a tracer experiment with [U-13C, U-15N] l-glutamine in the HCT116 cell line. The flow of the isotopic glutamine tracer is shown in Figure 3a. Incorporated [U-13C, U-15N] l-glutamine is metabolised to fumarate (m+4) and malate (m+4) in mitochondria through oxidative glutaminolysis and TCA cycle reactions. A portion of malate (m+4) becomes oxaloacetate (m+4) and citrate (m+4) through consecutive TCA cycle reactions in mitochondria. Another portion of malate (m+4) is exported to the cytoplasm from mitochondria and is converted into pyruvate (m+3) by ME1. Pyruvate (m+3) is converted into acetyl-CoA (m+2) in mitochondria, which then generates citrate (m+2) or citrate (m+6) (Figure 3a). In the experiment, we used ME1-1 siRNA, which suppressed ME1 expression but not cell growth 96 h after transfection, as a control to exclude metabolic changes caused by cell growth inhibition. Malate (m+4) and fumarate (m+4) were found to be accumulated after ME1 knockdown. Although pyruvate (m+3) levels were not reduced, both citrate (m+2) and citrate (m+6) levels decreased, whereas citrate (m+4) levels did not change (Figure 3b and Supplementary Figure S2A). These results suggest that the supply of pyruvate (m+3) and subsequently of acetyl-CoA (m+2) are reduced after ME1 knockdown without affecting TCA cycle reactions. We conclude that ME1 knockdown suppresses malate levels and its downstream metabolism in the HCT116 cell line.

### ME1 inhibition induces metabolic reprograming and disturbs redox homeostasis in cancer cell lines

**Figure 4:**
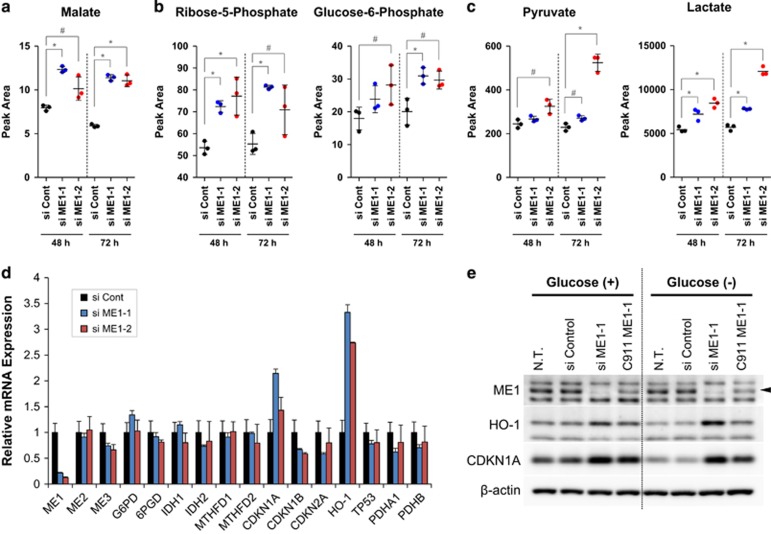
ME1 knockdown disturbs metabolic homeostasis and induces oxidative stress response in cancer cells. (**a**–**c**) Metabolomics analysis was conducted 48 and 72 h after ME1 siRNAs transfection in HCT116 cells. Peak area of malate, ribose-5-phosphate, glucose-6-phosphate, pyruvate and lactate are shown in the dot graph (*n*=3, *t*-test; *P<0.01; ^#^*P*<0.05). (**d**) Gene expressions of NADPH-producing enzymes, p53-related enzymes, a redox-related gene, and glycolysis-relating enzymes were detected by TaqMan PCR 48 h after ME1 siRNAs transfection in PC3 cells (*n*=3). (**e**) ME1, HO-1 and β-actin were detected by western blotting 96 h after control siRNA, ME1 siRNAs, or ME1 C911 transfection in HCT116 cells (ME1 is indicated by a black arrow). After siRNA transfection, cells were cultured in 2 g/l glucose McCoy's 5A media for 72 h, cultured in media with/without glucose for further 24 h, and then the cell lysate was collected for western blotting.

To investigate the effect of ME1 inhibition on cellular metabolism, we conducted metabolomics analysis in HCT116 cell lines after ME1 knockdown. We detected some metabolite level changes 48 h or 72 h after siRNAs transfection. Consistent with isotope profiling experiments (Figure 3b), malate and fumarate levels increased after ME1 depletion (Figure 4a and Supplementary Figure S2B). Intriguingly, the levels of glucose-6-phosphate (G6P) and ribose-5-phosphate (R5P), which are metabolites in PPP, also increased after ME1 depletion (Figure 4b). Furthermore, pyruvate and lactate levels increased significantly after ME1 inhibition (Figure 4c). These results suggest that glycolysis and PPP are enhanced as a result of ME1 inhibition in HCT116 cells. Because ME1 inhibition induced cellular metabolic reprograming, we speculated that ME1 depletion induces some cellular response. Therefore, we investigated the expression of NADPH-producing enzymes and senescence-related genes in HCT116 and PC3 cells. ME1 depletion increased CDKN1A and HMOX-1 (HO-1) mRNA levels (without affecting expression of other NADPH-producing enzymes in the PC3 cell line, Figure 4d), and the same result was observed in ME1-depleted HCT116 cells (Supplementary Figures S2C and S2D). To verify that CDKN1A and HO-1 expression were induced by ME1 inhibition, we examined CDKN1A and HO-1 protein expression in HCT116 cells using C911ME1 siRNA,^16^ which is similar in sequence to ME1 siRNA but was designed to remove gene knockdown ability, as a negative control. We also examined whether the presence of glucose could affect expression of CDKN1A and HO-1. The results show that CDKN1A and HO-1 protein levels increased after ME1 inhibition and the phenotypes were reverted by C911 (Figure 4e), suggesting that ME1 knockdown stimulates CDKN1A and HO-1 expression in HCT116 cells. Furthermore, we found that HO-1 expression was enhanced in glucose-depleted condition but that of CDKN1A was not (Figure 4e). These results suggest that ME1 inhibition disturbs redox homeostasis by regulating glucose metabolism.

In the H460 cell line, which shows high levels of ME1 expression (Figure 1b), HO-1 expression was enhanced and cell growth was inhibited by ME1 inhibition as observed in HCT116 and PC3 cells (Supplementary Figures S2E and S2F), although we did not observe senescence-like cells even in a clonogenic assay (data not shown). On the other hand, caspase-3,7 activity was augmented by ME1 knockdown in H460 cell lines, indicating that ME1 inhibition induced apoptosis in H460 cells (Supplementary Figure S2G). These results suggest that ME1 inhibition disturbs cellular metabolism and redox balance in several cancer cell lines but the specific phenotype, such as senescence or apoptosis, varies in each cell line.

### Glucose depletion increases reductive carboxylation and enhances ME1-dependent glutamine metabolism in HCT116 cells

**Figure 5:**
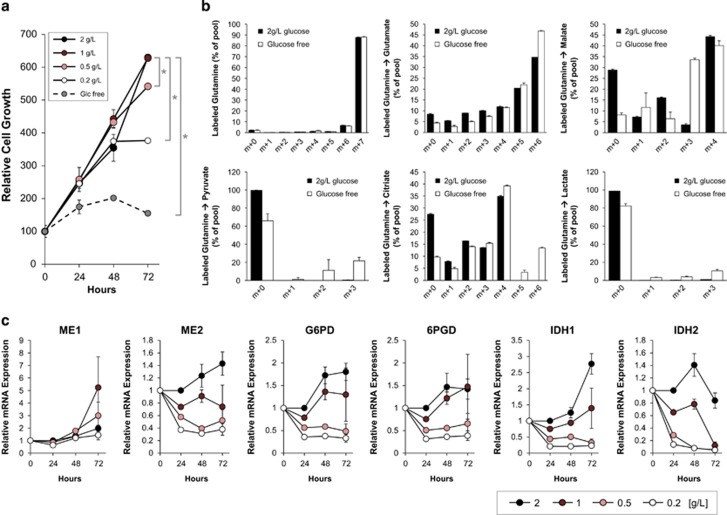
Glucose depletion increases ME1-dependent glutamine metabolism and reduces expression of NADPH-producing enzymes other than ME1 in HCT116 cells. (**a**) HCT116 cells were cultured in glucose-depleted or -limited media (2, 1, 0.5 or 0.2 g/l glucose or glucose-free) for 72 h. Cell viability was determined at 24, 48, and 72 h using CellTiter Glo. Relative cell growth, normalised by cell viability at Day 0, is shown in the line graph (*n*=3, *t*-test; *P<0.01). (**b**) HCT116 cells were cultured in media with or without glucose for 24 h and labelled with [U-13C, U-15N] l-glutamine isotope for an additional 24 h. Then, isotope profiling analysis was conducted. Percentage of the pool of metabolites and isotopes is shown in the bar graph (2 g/l glucose in black and glucose-free in white; *n*=3, mean with s.d.). (**c**) HCT116 cells were cultured in media with 2, 1, 0.5 or 0.2 g/l glucose for 72 h and mRNA expression levels of NADPH-producing enzymes at 0, 24, 48 and 72 h were determined by TaqMan PCR and are shown in the line graph (*n*=3).

We found that ME1 inhibition affected glucose metabolism, and that glucose concentration in the medium affected HO-1 expression induced by ME1 inhibition in HCT116 cells. Therefore, we investigated whether glucose availability affected malate metabolism and expression. We first conducted cell growth assays in glucose-restricted conditions. HCT116 cells were cultured in RPMI1640 media with 0, 0.2, 0.5, 1, and 2 g/l glucose, and cell viability was measured after 24, 48 and 72 h (normal medium contains 2 g/l glucose). Compared to 2 g/l glucose, HCT116 cell growth was suppressed after 48 h in 0.2 g/l, slightly suppressed after 72 h in 0.5 g/l, and suppressed in less than 24 h in glucose-free (0 g/l) medium (Figure 5a).

To monitor metabolic alterations in cells in glucose-depleted conditions, we performed a tracer experiment with [U-13C, U-15N] l-glutamine in the presence or absence of glucose in the medium. No difference in glutamine incorporation or malate (m+4) content was found; however, pyruvate (m+3), citrate (m+6) and lactate (m+3) levels increased (Figure 5b), suggesting that malate (m+4) metabolism is enhanced in glucose-depleted conditions. Furthermore, malate (m+0) and citrate (m+0) levels decreased in glucose-free conditions, suggesting that glucose-derived metabolites are reduced in cells. Malate (m+3) levels increased in glucose-free conditions, which may be due to activation of reductive carboxylation of glutamine. Reductive carboxylation produces citrate (m+5) from glutamine (m+7) and results in production of malate (m+3) through TCA cycle reactions (Figure 3a). Production of malate (m+3) results in an increase in downstream metabolites such as pyruvate (m+2) and lactate (m+2) (Figure 5b).

To examine the effects of glucose-restricted conditions on the expression of NADPH-producing enzymes, mRNA expression levels of ME1, ME2, G6PD, 6PGD, IDH1 and IDH2 were determined in HCT116 cells cultured in glucose-restricted media for 24, 48 and 72 h. Expression levels of NADPH-producing enzymes except ME1 decreased in a glucose concentration-dependent manner, whereas ME1 expression levels remained unchanged regardless of glucose concentration. These results suggest that HCT116 cells depend in part on ME1 in glucose-restricted conditions (Figure 5c). We conclude that glucose depletion induces reductive carboxylation instead of glycolysis and enhances ME1-dependent glutamine metabolism in HCT116 cells.

### ME1 knockdown synergistically suppress HCT116 cell growth in glucose-depleted conditions

**Figure 6:**
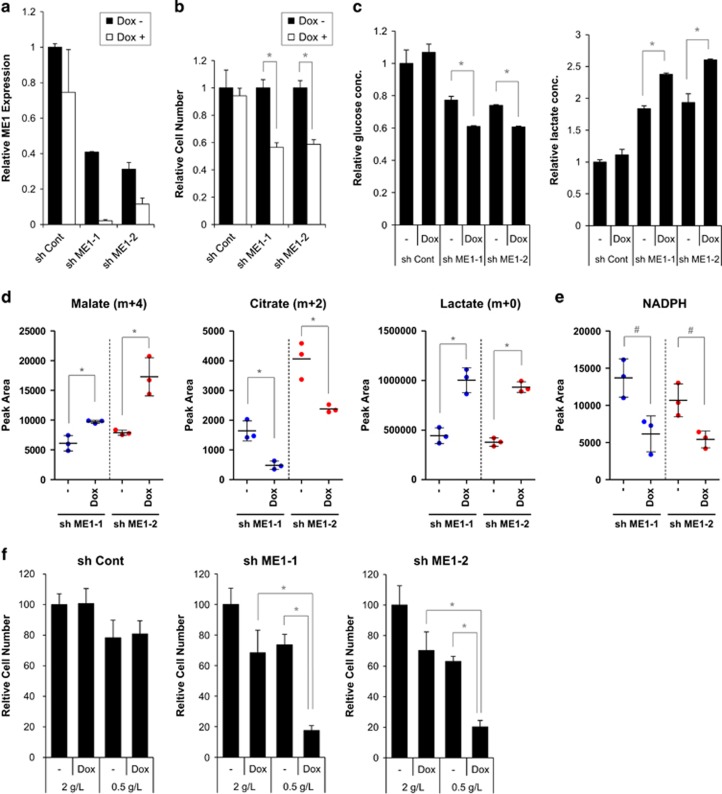
ME1 knockdown by shRNA induces metabolic reprograming and synergistically suppresses cancer cell growth in glucose-depleted conditions. HCT116 cell clones (sh ME1-1 and sh ME1-2), which are stably expressing tetracycline-inducible ME1 shRNA, were established. (**a**) Control shRNA, sh ME1-1 or sh ME1-2 clones were cultured in the media with or without 2 μg/ml doxycycline for 48 h, and ME1 expression was detected by TaqMan PCR. ME1 expression was normalised by GAPDH expression and relative ME1 expression is shown in the bar graph (*n*=3). (**b**) Cell numbers were determined 72 h with or without 2 μg/ml doxycycline. Relative cell number is shown in the bar graph (*n*=3, mean with s.d., *t*-test; *P<0.01) (**c**) Control shRNA, sh ME1-1 or sh ME1-2 were cultured in the media with or without 2 μg/ml doxycycline for 96 h and, d-glucose and l-lactate concentration in the media were measured. Relative glucose or lactate concentration are shown in the bar graph (*n*=3, mean with s.d., *t*-test; *P<0.01). (**d**,**e**) [U-13C, U-15N] l-glutamine isotope profiling analysis was conducted in sh ME1-1 and sh ME1-2 clones. Clones were cultured in the media with 2 g/l glucose with or without doxycycline for 72 h and labelled for additional 24 h in the glucose-depleted media with or without doxycycline. Then, metabolites and isotopes were measured. Peak area of labelled metabolites are shown in the dot graphs (*n*=3, *t*-test; *P<0.01; ^#^*P*<0.05). (**f**) Three clones were cultured for 24 h in 2 g/l medium with or without doxycycline, and additionally cultured for 72 h in medium with 2 or 0.5 g/l glucose with or without doxycycline. Then, cell numbers were determined by cell counter (*n*=3, mean with s.d.; *t*-test; *P<0.01).

Our experiments suggested that malate metabolism and glycolysis are coordinately regulated to maintain cellular redox homeostasis. We next examined whether ME1 inhibition synergistically suppressed cell growth in glucose-depleted conditions. We initially established shRNA tetracycline-inducible HCT116 cell clones, which expressed different shRNA sequences (sh control, sh ME1-1 or sh ME1-2) when induced by doxycycline. ME1 mRNA levels were suppressed 48 h after doxycycline treatment in HCT116 clones (Figure 6a). We also examined ME1 protein expression in sh control, sh ME1-1 and sh ME1-2 clones cultured for 48 h or 72 h with or without doxycycline in 2 g/l or 0.5 g/l glucose. ME1 expression was suppressed by ME1 shRNA regardless of glucose concentration (Supplementary Figures S3A and S3B). In contrast, ME2 mRNA expression levels remained unchanged after doxycycline treatment (Supplementary Figure S3C). Cell proliferation was partially suppressed by ME1 shRNA induction in HCT116 clones (Figure 6b). We determined d-glucose and l-lactate concentrations in the media in which sh control, sh ME1-1 or sh ME1-2-transfected cells were cultured for 96 h with or without doxycycline. Statistically significant decrease in D-glucose levels and increase in l-lactate levels were observed after ME1 shRNA induction (Figure 6c), suggesting that glycolysis is activated by ME1 inhibition in these clones. We next conducted a tracer experiment with [U-13C, U-15N] l-glutamine in HCT116 clones under glucose-depleted conditions. We initially determined if doxycycline affected cellular metabolism in the sh control clone, and found that doxycycline did not affect metabolite levels in glucose-sufficient or -depleted conditions (Supplementary Figure S3E). In sh ME1-1 and sh ME1-2 clones, accumulation of malate (m+4) and lactate (m+0) and reduction of citrate (m+2) were observed in both clones after ME1 knockdown (Figure 6d and Supplementary Figure S3D), indicating that malate metabolism is suppressed and glycolysis is activated in these clones. We also observed significant reduction in NADPH levels after ME1 knockdown in sh ME1 clones (Figure 6e), suggesting that redox balance is disturbed in these clones. To investigate the effect of ME1 depletion on cell growth in glucose-depleted conditions, we conducted cell growth assays with these clones under these conditions. HCT116 clones were treated with or without doxycycline and cultured for 24 h in normal culture conditions. Clones were then cultured for 96 h in normal (2 g/l) or 0.5 g/l glucose medium. ME1 knockdown significantly suppressed cell growth in glucose-restricted compared to normal culture conditions, indicating that glucose depletion and ME1 inhibition have a synergistic effect in HCT116 cell clones (Figure 6f and Supplementary Figure S4C). Similar results were observed in an additional clone (Supplementary Figures S4A and S4B). In contrast, glucose-restricted conditions did not synergistically suppress cell growth of a non-ME1 silencing control clone (Figure 6f and Supplementary Figure S4C). These results suggest that ME1 knockdown synergistically inhibits HCT116 cell growth in glucose-depleted condition.

## Discussion

NADPH production is regulated by multiple metabolic pathways to maintain cellular energy, reducing equivalents and biomolecules. Cancer cells regulate NADPH homeostasis through multiple pathways such as the PPP and the flux through ME1 and IDH1 in the cytoplasm. Recently, stepwise oxidation of one-carbon-tetrahydrofolate species was also reported to have a role in cellular NADPH production.^17^

ME1 metabolism is known to be regulated by well-known oncogenes or tumour suppressors such as K-ras, myc or p53.^7, 13^ Furthermore, ME1 is reported to be a potential prognostic or sensitivity marker of radio therapy.^11, 12^ Our immunocytochemis study revealed that ME1 localised in the cytosol, whereas ME2 and ME3 localised in the mitochondria, which is consistent with previous findings.^8, 9^ We found that ME1 is highly expressed in most cancer cell lines compared to ME2 and ME3, although some cell lines predominantly expressed ME2 or ME3. Taken together, these findings suggest that ME1 has an important function in the growth and survival of cancer cells and that it could be a target for cancer therapy.

The ME1 knockdown experiments described here revealed that ME1 depletion does not induce acute cell growth inhibition, but suppresses cancer cell growth gradually by inducing senescence in HCT116 and PC3 cell lines or apoptosis in H460 cell line. Partial cell growth inhibition was also observed in other cancer cell lines (Supplementary Table 1). We demonstrated that ME1 knockdown increased the expression of HMOX-1 (HO-1), which is an oxidative stress marker regulated by Nrf2,^18^ suggesting that ME1 depletion disturbs metabolic and redox balance in cancer cells. We speculate that ME1 depletion suppresses cancer cell growth by abrogating metabolic and redox balance in the cancer cells, and the specific phenotype such as senescence, apoptosis, or others, depends on cellular context in each cell line. We need further studies to identify cell fate determinants in ME1-suppressed cancer cells, which could be sensitivity markers for a ME1 inhibitor in clinical settings.

l-glutamine isotope profiling analysis revealed the metabolic alterations in cancer cells by ME1 knockdown. Accumulation of malate (m+4) and fumarate (m+4) implies inhibition of the malate flux by ME1 knockdown. Reduction in citrate (m+2) and citrate (m+6) levels, but not in citrate (m+4) levels, indicates that the malate-pyruvate-acetyl-CoA flux is also suppressed by ME1 knockdown. Although pyruvate (m+3) reduction was expected after ME1 knockdown, no significant changes in pyruvate levels were observed. This may be due to pyruvate (m+3) being immediately converted to acetyl-CoA and the malate-to-pyruvate conversion being rate-limiting in cells. Basal pyruvate levels would thus be kept low, and a reduction would be difficult to detect.

Pyruvate accumulation was observed in our metabolomics analysis (Figure 4c). We also observed lactate accumulation by ME1 siRNA (Figure 4c) and ME1 shRNAs (Figure 6d), which may be caused by upregulation of glycolysis. Increase of R5P and G6P levels supports this hypothesis (Figure 4b). We also found that glucose levels decreased and lactate levels increased in the media after ME1 knockdown in HCT116 cells (Figure 6c), suggesting that the Warburg Effect is enhanced by ME1 inhibition in HCT116 cells. We demonstrated that ME1 knockdown induced HO-1 expression, a marker of oxidative stress (Figures 4d and e). Cancer cells may enhance glycolysis after ME1 depletion to compensate for the reduction in NADPH by activating PPP in the cytoplasm and may adapt to an oxidative state for protection. HO-1 expression after ME1 inhibition was higher in glucose-depleted compared to glucose-sufficient conditions (Figure 4e), suggesting that glycolysis may relieve the oxidative state in HCT116 cells. Alternatively, because pyruvate is known to have antioxidative function,^19, 20^ cancer cells may manage the oxidative state by increasing pyruvate levels in addition to the activation of the PPP pathway.

Although both si ME1-1 and si ME1-2 suppressed ME1 expression at mRNA and protein levels (Supplementary Figures S1A and S1B) and both enhanced malate accumulation (Figure 4a), which is a target engagement marker of ME1 inhibition, si ME1-2 showed stronger cell growth inhibition than si ME1-1. We believe that si ME1-2 has an off-target effect in addition to ME1 inhibition. Enhanced accumulation of pyruvate and lactate by si ME1-2 compared to si ME1-1 may be caused by this off-target effect.

We demonstrated that ME1 inhibition induces metabolic reprogramming and abrogates redox balance in cancer cells, both being related to glucose metabolism. Therefore, we investigated if glucose concentration in the media could affect cellular malate metabolism and ME levels. Isotope profiling analysis indicated that cellular metabolic balance is changed from glycolysis-dependent to glutaminolysis-dependent in glucose-depleted conditions (Figure 5b). Malate (m+0) levels decreased in glucose-free conditions because of the reduction in glucose-derived metabolites. Decrease in citrate (m+0) levels supports this hypothesis. The increase in malate (m+3) levels in glucose-free conditions may be due to activation of reductive carboxylation of glutamine. Reductive carboxylation produces citrate (m+5) from glutamine (m+7) and results in the production of malate (m+3) through TCA cycle reactions. Increase of malate (m+3) results in increase of pyruvate (m+2) and lactate (m+2). Malate (m+4) levels are similar between normal glucose and glucose-free conditions. However, pyruvate (m+3) and citrate (m+6) were observed only in cells cultured in glucose-free media, suggesting that ME1 activity could be induced in cancer cells under glucose-depleted condition. ME1 expression did not decreased in glucose-depleted conditions, although the levels of other NADPH-producing enzymes decreased (Figure 5c), which suggests that cancer cells depend in part on ME1 in glucose-depleted conditions. Here, we demonstrated that HCT116 cells increase its dependency on ME1 metabolism in glucose-depleted conditions.

From these results, we speculated that ME1 inhibition would synergistically inhibit cancer cell growth in glucose-depleted conditions. To address the hypothesis, we established ME1 shRNA-inducible HCT116 cell lines, which showed similar metabolic changes to HCT116 cells transfected with ME1 siRNA under ME1 shRNA induction in glucose-depleted conditions (Figure 6d). Expression of ME1 mRNA in sh ME1 clones were lower than that of sh control clones, which may be due to leak of ME1 shRNA in the clones. These results may also explain the higher glucose consumption and higher lactate production in ME1 shRNA clones than those in sh control clones (Figures 6a and c). We demonstrated that combining ME1 inhibition with glucose depletion synergistically inhibited cancer cell growth. We did not observe any cells with senescence-like or apoptotic phenotypes in these cells. Senescence-like phenotype similar to that shown in Figure 2d could be observed if shRNA-expressing cells were cultured for a much longer period in normal media. However, cells died earlier in glucose-depleted than in glucose-sufficient conditions, suggesting that another cell killing mechanism was activated by combining ME1 inhibition and glucose depletion. The oxidative state, indicated by a reduction in NADPH levels, may result in cell growth inhibition. Additional studies such as rescue of growth inhibition by antioxidants would test this hypothesis.

An *in vivo* efficacy study is needed to verify whether ME1 inhibition is effective as a tumour growth suppressor. It is known that glucose levels are depleted within the tumour, although the tumour-induced angiogenesis, which provides nutrients (including glucose) to support rapid growth, may lead to higher levels of intra-tumour glucose than expected. Therefore, the efficacy of ME1 inhibition on tumour growth suppression probably depends on the tumour microenvironment, and *in vivo* experiments are necessary to clarify the issue. We hypothesise that a treatment strategy combining an ME1 inhibitor with an inhibitor of glycolysis, such as 2-DG, or with an inhibitor of glucose transporter, such as GLUT-4 inhibitor, could result in a synergic action against the tumour regardless of tumour microenvironment.

ME1 inhibition may be toxic in some tissues, as ME1 is ubiquitously expressed. Tumours sensitive to ME1 inhibition should therefore be selected. For example, ME1 expression is known to be regulated by Nrf2, and tumours with mutated *Keap1* or *Nrf2* genes could be dependent on ME1 and thus be sensitive to ME1 inhibition. Alternatively, as described above, a glucose-depleted microenvironment could make tumours sensitive to ME1 inhibition.

In this study, we demonstrated that ME1 is an essential enzyme that regulates cellular metabolism and redox balance. We also showed that, in glucose-depleted conditions, cancer cells become dependent on the ME1 flux to produce NADPH and pyruvate and to manage redox homeostasis, suggesting that these cells become vulnerable to reduced ME1 activity. Taken together, our results demonstrate that ME1 inhibition suppresses cancer cell growth and induces apoptosis or senescence depending on the cellular context, consistent with previous reports,^7, 13, 14^ and that tumours in a nutrient-limited microenvironment are a sensitive target for ME1 inhibition. No ME1 inhibitors are currently being evaluated in clinical trials, although a few small-molecule ME2 inhibitors were discovered in pre-clinical trials.^21, 22, 23^ A treatment strategy combining ME1 inhibitors with inhibition of glycolysis, NADPH-producing enzymes, or redox-regulating enzymes would provide an effective therapeutic option for some types of cancer. Further work is required in animal models to assess if such a strategy would be effective in tumours.

## Materials and methods

### Cell lines and culture

Human cancer cell lines were purchased from American Type Culture Collection (Manassas, VA, USA). HCT116 cells, HCT116 shRNA-expressing stable cell clones and PC3 cells were cultured in RPMI1640 (Cat. No. 22400-089, GIBCO/ThermoFisher Scientific, Waltham, MA, USA) with 10% FBS (Hyclone/GE healthcare, Chicago, IL, USA). Glucose-free or glutamine-free RPMI1640 medium (Cat. No. 11879020, Cat. No. 42401-018, GIBCO/ThermoFisher Scientific) and Glucose- and glutamine-free RPMI1640 medium (obtained based on the components of RPMI1640 by Funakoshi, Tokyo, Japan) were used for isotope profiling analysis and growth assays. Tetracycline-free FBS (Clonetech/Takara Bio USA, Inc., Mountain view, CA, USA) were used with shRNA-expressing cell clones, and dialysed FBS (Cat. No. 26400-044, GIBCO/ThermoFisher Scientific) were used for isotope profiling analysis. H460 cells were cultured in DMEM medium (Cat. No. 11965-092, GIBCO/ThermoFisher Scientific).

### siRNAs, shRNAs and reagents

SilencerSelect siRNAs, SilencerSelect Negative Control No. 2 (439080846), KIF11 (s7903), ME1 (s8637, s8639), and ME2 (s8640, s8642) were purchased from Ambion/ThermoFisher Scientific (Waltham, MA, USA). C911 siRNA was ordered and synthesised by Ambion. The C911 siRNA sense and antisense sequences are 5′-CCAGGUUCAAUGAGUAGUAtt-3′ and 5′-UACUACUCAUUGAACCUGGat-3′, respectively. Doxycycline hyclate was purchased from Sigma-Aldrich (Cat. No. D9891-5G, St Louis, MO, USA) and crystal violet from Wako Chemicals GmbH (Cat. No. 038-04862, Neuss, Germany). pTRIPZ-inducible lentiviral shRNAs for non-silencing control and ME1 were purchased from Open Biosystems/GE healthcare, (Cat. No. RHS4743, Clone IDV2THS_151673, V3THS_322622, Chicago, IL, USA).

### siRNA transfection

For siRNA experiments, cells were reversely transfected with siRNAs using Lipofectamine RNAiMAX transfection reagent (Invitrogen, Waltham, MA, USA) according to the manufacturer's recommendations. Individual siRNAs were transfected at a final concentration of 10 nm.

### Clonogenic assay

HCT116 cells were seeded in six-well plates at 1000 cells/well and were reversely transfected with siRNAs. After siRNA transfection, cells were cultured for 11 days with medium changed every 3 days. Colonies were washed twice with phosphate-buffered saline, fixed with 4% formaldehyde for 15 min, and stained with crystal violet. Colony area was quantified by GelCount Tumour Colony Counter (Oxford Optronix, Abingdon, UK).

### Cell proliferation, cell count, caspase-3/7 and senescence analyses

Cell viability was determined using the CellTiter-Glo luminescence assay (Promega, Madison, WI, USA) according to the manufacturer's suggested protocol. Luminescent signals were detected using an ARVO MX1420 microplate reader (PerkinElmer, Wellesley, MA, USA). Cell numbers were determined using a TC10 automated cell counter (Bio-Rad Laboratories, Hercules, CA, USA). In general, cancer cells were reversely transfected with siRNA and cell proliferation or cell numbers were determined 96 h after siRNA transfection. Caspase-3/7 activity was determined using the Caspase-Glo 3/7 Assay (Promega). Cellular senescence was assessed using the SA-b-Gal Staining kit from Cell Biolabs (Cat. No. CBA-230, San Diego, CA, USA).

### Glucose and lactate assays

Glucose and l-lactate concentrations in the medium were determined using LabAssay Glucose (Wako Chemicals GmbH) and l-Lactate Assay Kit (Colorimetric) (Abcam, Cambridge, UK), respectively.

### Establishment of stable pTRIPZ ME1 cell lines

HCT116 cells were transfected with pTRIPZ vectors and selected with 1 μg/ml puromycin 48 h after transfection. Cells were cultured in medium with 1 μg/ml puromycin for 14 days and grown colonies were picked up. Then, stably shRNA-expressing HCT116 cell clones were established.

### Metabolomics and isotope profiling analyses

After siRNA or shRNA treatment, 0.5–1.0 × 10^6^ cells were suspended in 1 ml of 100% methanol and centrifuged for 5 min at 15 000 *g* at 4 °C. The supernatant (200 μl) of cell extracts was dried by nitrogen stream and then derivatised by a two-step reaction: oximation and trimethylsilylation. The reaction mixture (1 μl) was injected into an Agilent 7890 A/5975C gas chromatography-mass spectrometer (GC/MS) (Agilent Technologies, Santa Clara, CA, USA) equipped with a J&W Scientific HP-5MS column (30 m × 0.25 mm i.d., film thickness=0.25 μm, Agilent Technologies), and molecules were separated in a temperature gradient from 60 °C to 325 °C at 10 °C/min. The GC/MS data were analysed using ChemStation software (Agilent Technologies), and the peak areas of target molecules were exported to a spreadsheet for further analysis. For stable isotope tracer analysis, the abundances of mass isotopologues were quantified by subtracting the abundance of natural isotopes from the raw mass spectra.^24^

### Statistical analyses

All data represented the results from three independent experiments. The statistical differences between groups were determined using a two-tailed Student’s *t*-test.
