# Supplementary material for: Inhibition of malic enzyme 1 disrupts cellular metabolism and leads to vulnerability in cancer cells in glucose-restricted conditions
Source: Oncogenesis. 2017 May 8;6(5):e329–. doi: 10.1038/oncsis.2017.34 (PMC5523067; doi:10.1038/oncsis.2017.34)
Supplement: Supplementary Figures Legends [file oncsis201734x1.docx]

**Supplementary Figure Legends**

**Supplementary Figure 1.** (A) ME1 and ME2 mRNA levels were normalised by GAPDH and are shown in the bar graph. HCT116 cells were reversely transfected with control, ME1, or ME2 siRNAs, and RNAs were collected 48 h after transfection. ME1 and ME2 expression were determined by TaqMan analysis (n=3). (B) Expression of ME1, ME2, and β-actin in HCT116 cells were detected by western blotting. Cell lysate was collected 48 h after transfection with the indicated siRNAs. (C) HCT116 cells were reversely transfected with indicated siRNAs for 96 h. Cell proliferation was determined by CellTiter Glo (n=3). (D) Expression of ME1 and β-actin in PC3 cells were determined by western blotting. Cell lysate was collected 48 h after transfection with the indicated siRNAs. (D) Light microscopy images of colonies of PC3 cells transfected with control and ME1 siRNAs and cultured for 9 days.

**Supplementary Figure 2.** (A) Additional metabolite graphs of the experiments described in Figure 3A (n=3, t-test; *, *p<0.01*; #, *p<0.05*). (B) Additional metabolite graphs of the experiments described in Figure 4A-4C (n=3, t-test; *, *p<0.01*; #, *p<0.05*). (C) Relative mRNA expression levels of NADPH-producing enzymes under ME1 knocked down condition in HCT116 cells. mRNA levels were determined 48 and 72 h after siRNA transfection. Expression of indicated genes was normalised by GAPDH and relative expressions are shown in the bar graph (n=3). (D) CDKN1A and HO-1 mRNA expression levels in HCT116 cells were determined by TaqMan analysis. mRNAs were extracted from HCT116 cells 72 h after control and ME1 siRNAs transfection. Expression of CDKN1A and HO-1 was normalised by GAPDH and relative expression levels are shown in the bar graph (n=3). (E) Expression of ME1, HO-1, and β-actin detected by western blotting 96 h after control siRNA, ME1 siRNAs, or ME1 C911 transfection in H460 cells. After siRNA transfection, cells were cultured for 96 h and cell lysate was collected for western blotting. (F) Cell numbers were determined 96 h after siRNAs or C911 transfection in H460 cells. Cell number per mL is shown in the bar graph (n=3, t-test; #, *p<0.05*). (G) Caspase-3,7 activity was determined 96 h after siRNA or C911 transfection in H460 cells. Relative caspase-3,7 activity is shown in the bar graph (n=3, t-test; *, *p<0.01*; #, *p<0.05*).

**Supplementary Figure 3.** (A, B) ME1 and β-actin expression determined by western blotting in sh control, sh ME1-1 and sh ME1-2 clones. Clones were cultured with or without 2 µg/mL doxycycline for 48 h or 72 h in media with 2 g/L or 0.5 g/L glucose. (C) Relative ME2 expression determined in shRNA clones cultured with or without 2 µg/mL doxycycline for 48 h (n=3). (D) Additional metabolite graphs of the experiments described in Figures 6D and 6E (n=3, t-test; *, *p<0.01*). (E) Metabolomics analysis conducted in the sh control clone. The sh control clone was cultured in the media with 2 g/L glucose with or without doxycycline for 72 h and cultured for an additional 24 h in the media with 2 g/L glucose or glucose-free with or without doxycycline. Then, metabolites and isotopes were measured. Peak area of labelled metabolites are shown in the dot graphs (n=3).

**Supplementary Figure 4.** (A) Relative ME1 and ME2 expression levels determined in sh control and a ME1-2 shRNA clone (clone 65). The clones were cultured for 48 h with or without 2 µg/mL doxycycline and RNAs were extracted. mRNA expression levels were determined by TaqMan analysis and normalised by GAPDH mRNA levels (n=3). (B) sh control and clone 65 were cultured for 24 h in 2 g/L medium with or without doxycycline, and additionally cultured for 72 h in medium with 2 g/L or 0.5 g/L glucose with or without doxycycline. Cell numbers were determined by cell counter (n=3, t-test; *, *p<0.01*). (C) Light microscopy images of HCT116 sh control, sh ME1-1, sh ME1-2, and clone 65. The clones were cultured for 24 h in 2 g/L medium with or without doxycycline, and additionally cultured for 72 h in medium with 2 g/L or 0.5 g/L glucose with or without doxycycline.

**Supplementary Materials and Methods**

**Immunocytochemistry**

U2OS cells were cultured on Falcon 8 well culture slide (Corning, NY, USA) for 24 h and transfected with ME1, ME2 and ME3 cDNAs using Lipofectamine 2000 (Invitrogen). Forty-eight hours after transfection, cells were stained with MitoTracker Red CM-H2Xros (ThermoFisher Scientific) for 30 minutes and were fixed in 4% formaldehyde in PBS for 30 minutes at room temperature. Then, cells were washed with PBS, permeabilized with cold methanol for 30 minutes at -20 °C, and treated with StartingBlock (PBS) blocking buffer (ThermoFisher scientific) for 1 h. Cells were incubated with ME1 (sc-135303, Santa Cruz), ME2 (ab191693, Abcam) or ME3 (ab172972, Abcam) for 1 h and then secondary antibody (Alexa Fluor 488, Abcam). Finally, cells were mounted with VECTASHIELD mounting medium with DAPI (VECTOR laboratories, Inc. Burlingame, CA, USA) and observed by a fluorescent microscopy.

**FACS analysis**

For cell size measurement using forward scatter (FSC) with fixed cells, PC3 cells were centrifuged at 300 rpm for 3 min, washed twice with PBS, and suspended in 150 μL of cold PBS. A volume of 350 μL of cold 100 % ethanol was added to the cell suspensions, which were vortexed and incubated at -20 °C for 30 min. The cells were then centrifuged at 300 rpm for 3 min, resuspended in PBS containing 20 ng/μL RNase A (Sigma-Aldrich), and incubated at 25 °C for 30 min. Next, propidium iodide (PI) was added at a concentration of 40 ng/μL, and cells were incubated for 10 minutes at 25 °C. Samples were analysed by FACS to determine cell size (using FSC) and distribution in the cell cycle.

**Quantitative RT-PCR analysis**

Cancer cells were transfected with siRNAs and, 96 h after transfection, total RNA was isolated from cells and purified with the RNeasy Mini Kit (Qiagen, Valencia, CA, USA) according to manufacturer's instructions. Quantitative real-time PCR analysis was performed on a ViiA7 (Applied Biosystems, Foster City, CA, USA), using TaqMan Fast Advanced Master Mix with TaqMan probes against the indicated genes (Applied Biosystems). Data were analysed according to the 2^–ΔΔCt^ method and normalised relative to the amount of *GAPDH* mRNA. The normalised abundance of target mRNAs was expressed relative to the corresponding values for cells treated with dimethyl sulphoxide or negative control siRNAs. The following TaqMan probes were used for quantitative RT-PCR analysis: ME1 (Hs01554892_m1), ME2 (Hs00929809_g1), ME3 (Hs00198572_m1), G6PD (Hs00166169_m1), 6PGD (Hs00427230_m1), IDH1 (Hs01855675_s1), IDH2 (Hs00158033_m1), MTHFD1 (Hs01068263_m1) and MTHFD2 (Hs00759197_s1), CDKN1A (Hs00355782_m1), CDKN1B (Hs01597588_m1), CDKN2A (Hs00923894_m1), HMOX-1 (Hs01110250_m1), TP53 (Hs01034249_m1), PDHA1 (Hs01049345_g1), PDHB (Hs00168650_m1), GAPDH (Hs99999905_m1), and 18SrRNA (Hs99999901_s1).

**Western blotting**

Whole cell extracts were prepared with 1 × Laemmli sample buffer (Tris-HCl 125 mM pH 7.5, 1 % SDS, 20 % glycerol). The extracts were fractionated by SDS-PAGE, and the separated proteins were transferred using an iBlot Transfer Stack (Nitrocellulose) and an iBlot Gel Transfer Device (Invitrogen/Thermo Fisher Scientific, Waltham, MA, USA). After incubation with StartingBlockT20 (PBS) blocking buffer (Pierce Biotechnology, Rockford, IL, USA), the membranes were labelled for approximately 16 h with primary antibodies, followed by incubation with horseradish peroxidase-conjugated secondary antibodies (Cell Signaling Technology, Beverly, MA, USA). Membranes were incubated with ImmunoStar Zeta (Wako Chemicals GmbH) and scanned using an ImageQuant LAS-3000 (Fujifilm, Tokyo, Japan). The following antibodies were used for western blotting analysis: ME1 (H-47) (sc-135303) and actin (C-2) (sc-8432) from Santa Cruz, HO-1 (HMOX-1) (ADI-SPA-896) from Enzo Life Sciences (Farmingdale, NY, USA).
