# Supplementary figures and images for: Inhibition of malic enzyme 1 disrupts cellular metabolism and leads to vulnerability in cancer cells in glucose-restricted conditions
Source: Oncogenesis. 2017 May 8;6(5):e329–. doi: 10.1038/oncsis.2017.34 (PMC5523067; doi:10.1038/oncsis.2017.34)

## Slide 1
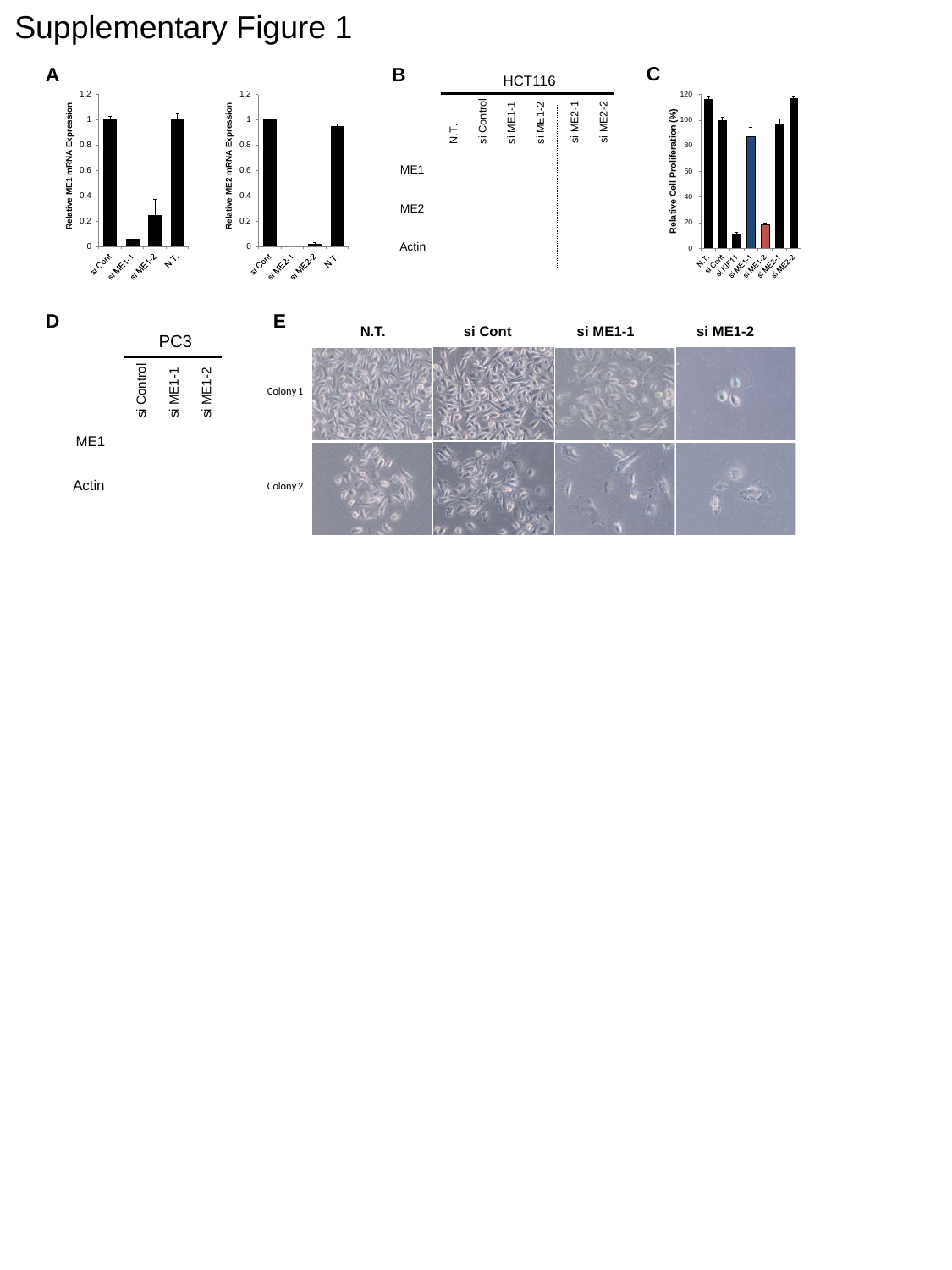

Supplementary Figure 1
C
A
B
D
E
si Cont
N.T.
si ME1-1
si ME1-2

Supplement: Supplementary Figure 1 [file oncsis201734x2.ppt]

## Slide 1
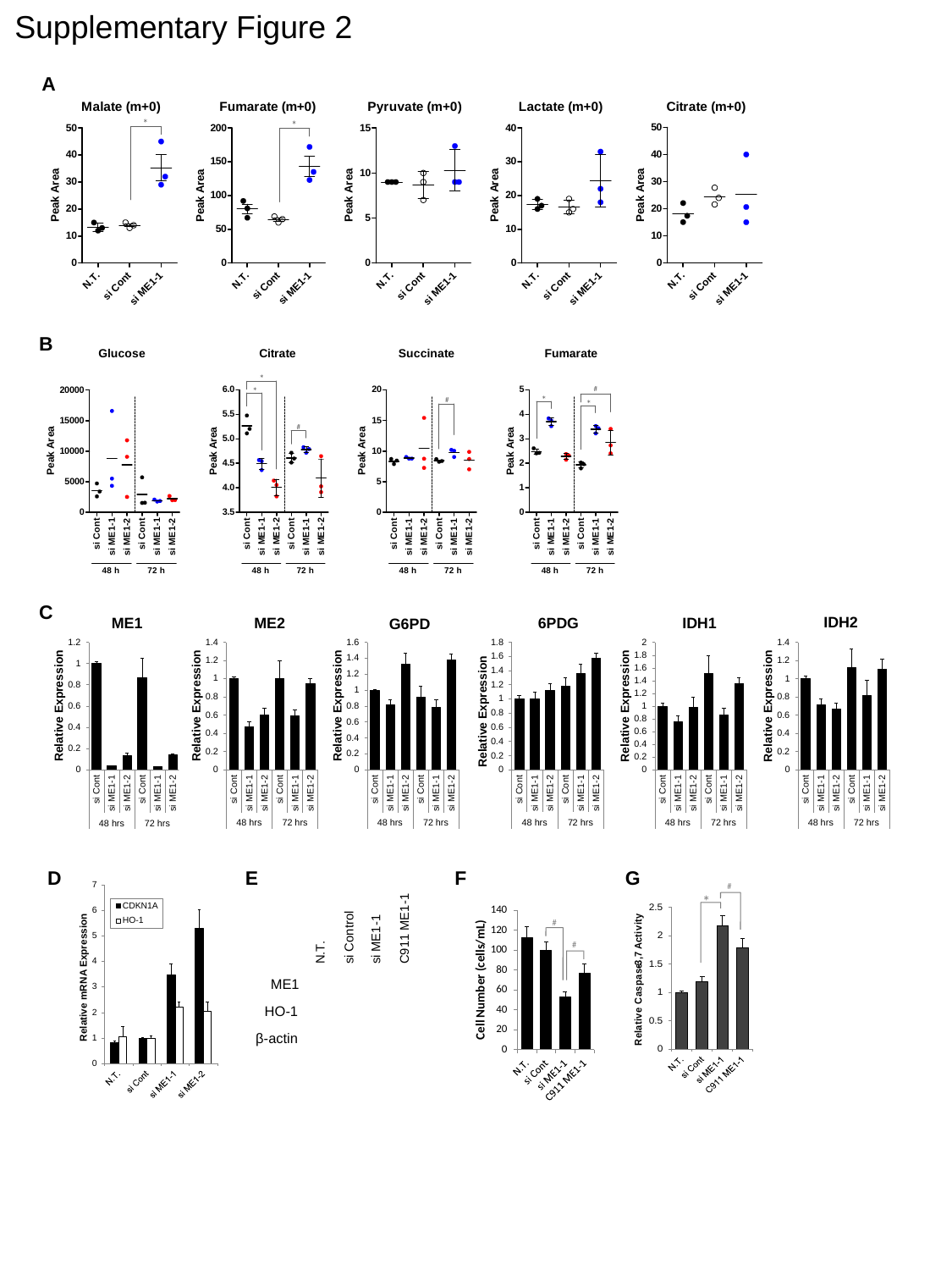

Supplementary Figure 2
A
B
C
D
E
F
G
#
*
#
#

Supplement: Supplementary Figure 2 [file oncsis201734x3.ppt]

## Slide 1
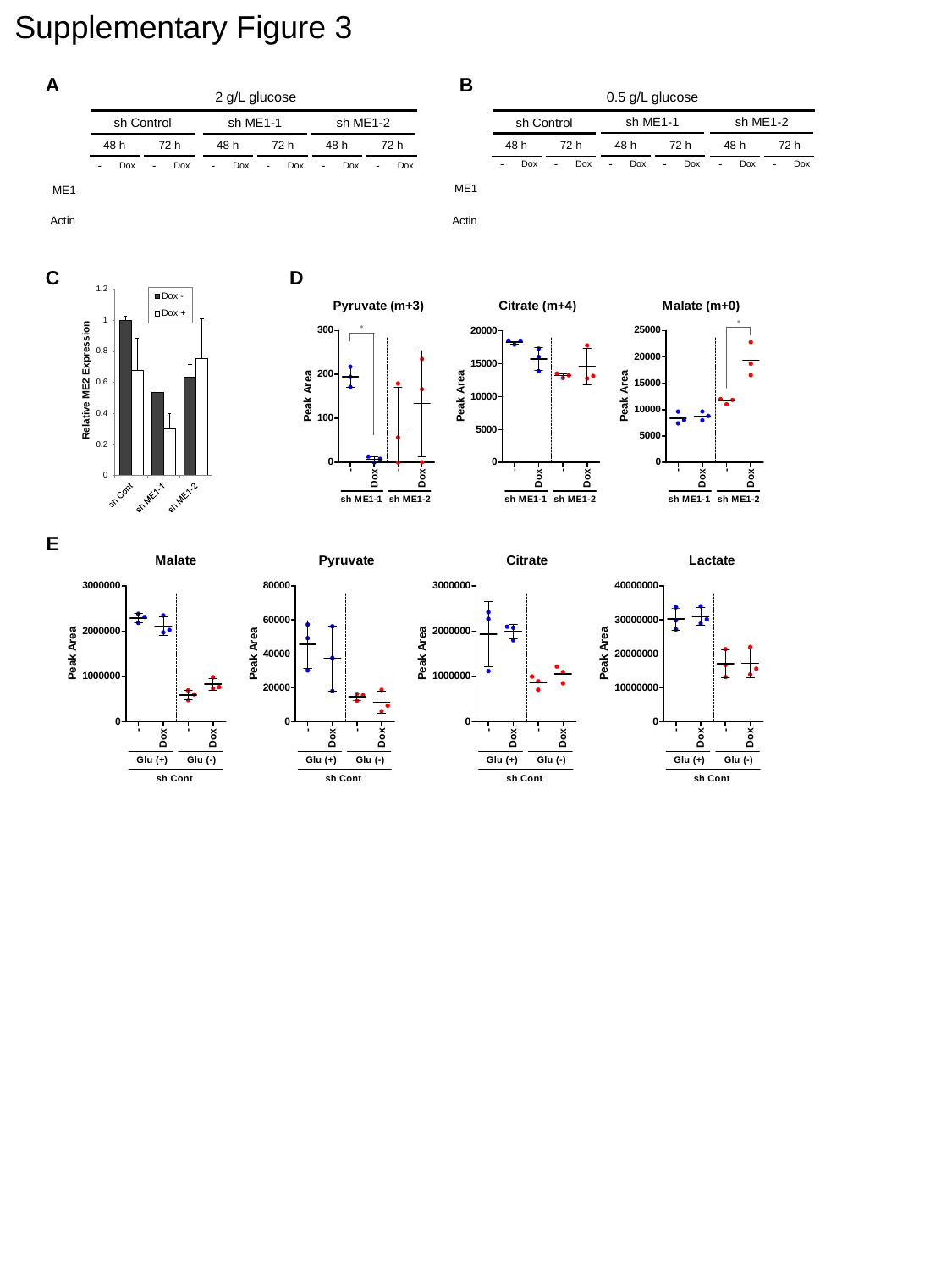

Supplementary Figure 3
A
B
C
D
E

Supplement: Supplementary Figure 3 [file oncsis201734x4.ppt]

## Slide 1
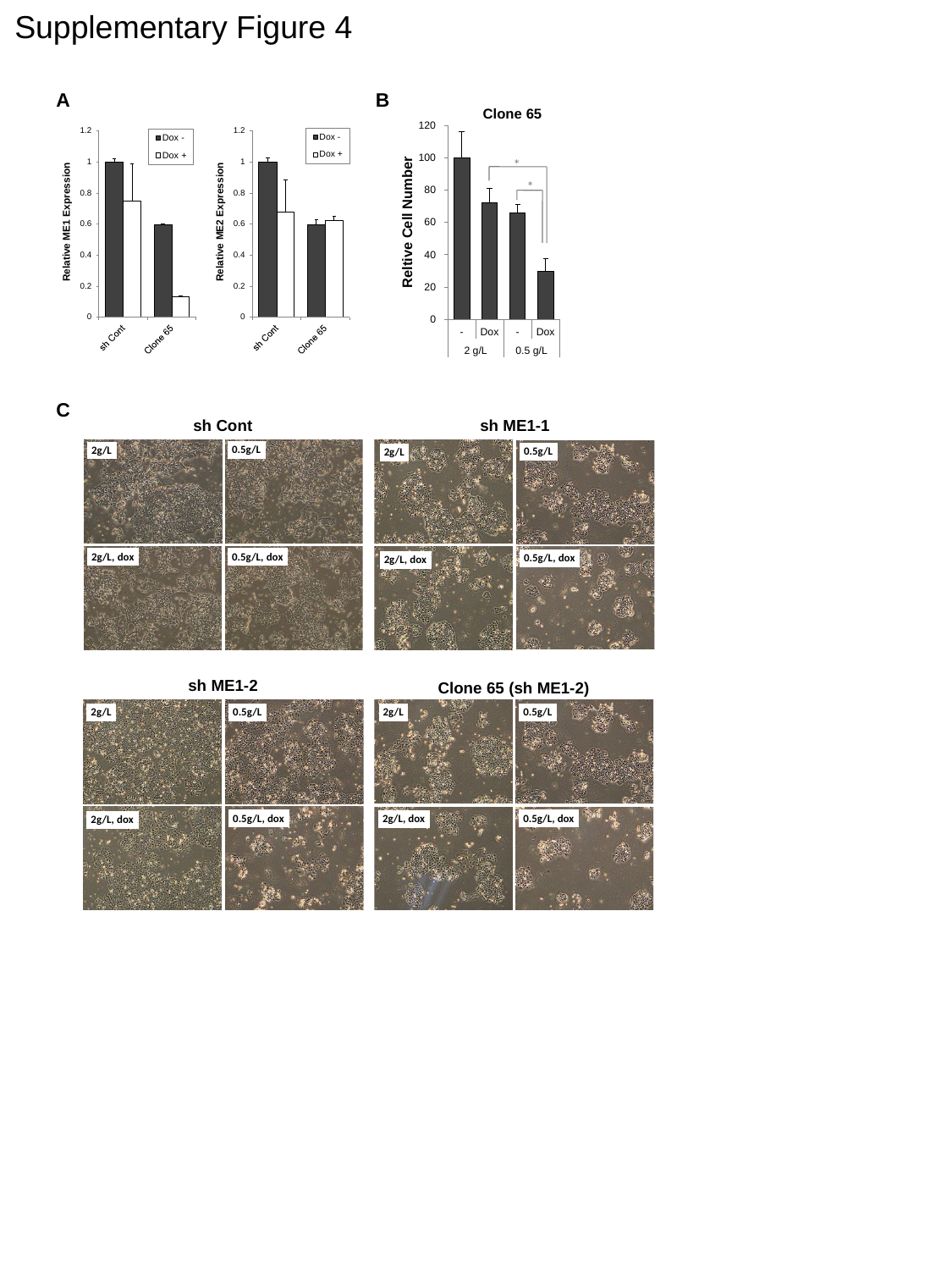

Supplementary Figure 4
A
B
Clone 65
*
*
C
sh Cont
sh ME1-1
sh ME1-2
Clone 65 (sh ME1-2)

Supplement: Supplementary Figure 4 [file oncsis201734x5.ppt]
