# Supplementary material for: Inhibition of malic enzyme 1 disrupts cellular metabolism and leads to vulnerability in cancer cells in glucose-restricted conditions
Source: Oncogenesis. 2017 May 8;6(5):e329–. doi: 10.1038/oncsis.2017.34 (PMC5523067; doi:10.1038/oncsis.2017.34)
Supplement: Supplementary Table 1 [file oncsis201734x6.docx]

**Supplementary Table 1.** Relative cell proliferation of cell lines 96 h after reverse transfection with siRNAs. Cells were cultured in media with normal glucose concentration.
